# Enhancing educational experience through establishing a VR database in craniosynostosis: report from a single institute and systematic literature review

**DOI:** 10.3389/fsurg.2024.1440042

**Published:** 2024-09-04

**Authors:** Attill Saemann, Sina Schmid, Maria Licci, Marek Zelechowski, Balazs Faludi, Philippe C. Cattin, Jehuda Soleman, Raphael Guzman

**Affiliations:** ^1^ Department of Neurosurgery, University Hospital of Basel, Basel, Switzerland; ^2^ Department of Biomedical Engineering, University of Basel, Allschwil, Switzerland; ^3^ Faculty of Medicine, University of Basel, Basel, Switzerland

**Keywords:** VR, craniosynostosis, pediatric neurosurgery, education, virtual reality, teaching

## Abstract

**Background:**

Craniosynostosis is a type of skull deformity caused by premature ossification of cranial sutures in children. Given its variability and anatomical complexity, three-dimensional visualization is crucial for effective teaching and understanding. We developed a VR database with 3D models to depict these deformities and evaluated its impact on teaching efficiency, motivation, and memorability.

**Methods:**

We included all craniosynostosis cases with preoperative CT imaging treated at our institution from 2012 to 2022. Preoperative CT scans were imported into SpectoVR using a transfer function to visualize bony structures. Measurements, sub-segmentation, and anatomical teaching were performed in a fully immersive 3D VR experience using a headset. Teaching sessions were conducted in group settings where students and medical personnel explored and discussed the 3D models together, guided by a host. Participants’ experiences were evaluated with a questionnaire assessing understanding, memorization, and motivation on a scale from 1 (poor) to 5 (outstanding).

**Results:**

The questionnaire showed high satisfaction scores (mean 4.49 ± 0.25). Participants (*n* = 17) found the VR models comprehensible and navigable (mean 4.47 ± 0.62), with intuitive operation (mean 4.35 ± 0.79). Understanding pathology (mean 4.29 ± 0.77) and surgical procedures (mean 4.63 ± 0.5) was very satisfactory. The models improved anatomical visualization (mean 4.71 ± 0.47) and teaching effectiveness (mean 4.76 ± 0.56), with participants reporting enhanced comprehension and memorization, leading to an efficient learning process.

**Conclusion:**

Establishing a 3D VR database for teaching craniosynostosis shows advantages in understanding and memorization and increases motivation for the study process, thereby allowing for more efficient learning. Future applications in patient consent and teaching in other medical areas should be explored.

## 1 Introduction

Virtual Reality (VR) has emerged as a transformative force in medical education, offering a paradigm shift in the pedagogical approach, particularly within neurosurgery. Integrating VR technology presents an opportunity to broaden the educational landscape within the confines of neurosurgical education, where the demand for an in-depth understanding of intricate anatomical structures and precise surgical techniques is paramount (1, 2). Multiple studies have discussed virtual reality's pivotal role in neurosurgical training, emphasizing its capacity to provide an immersive, experiential learning environment that goes beyond traditional didactic methods (3–5).

Traditional instructional modalities, such as textbooks and cadaveric dissections, fall short of delivering a truly immersive educational experience (6). As an innovative teaching tool, VR offers the potential to transcend these limitations by offering a dynamic, three-dimensional environment wherein students and practitioners can navigate and interact with realistic anatomical structures, facilitating a deeper understanding of the complexities (4, 7). Additionally, the use of VR has been shown to increase the learner's motivation, which has a proven positive effect on learning productivity (8).

The application of VR in neurosurgical education extends beyond visual representation; VR platforms allow learners to engage in group teaching, practice surgical procedures, and maneuver through authentic anatomical structures, all within a simulated and thus risk-free and resource-saving environment (9, 10). This augments their comprehension of neurosurgical intricacies and facilitates repetitive practice, which is crucial for developing a profound three-dimensional anatomical understanding (9, 10). In addition to the educational use, a clinical application of VR across the neurosurgical treatment continuum is being established. Many studies are investigating its use in presurgical planning, resident training, and patient education (11–15).

VR is potentially particularly beneficial for highly variable and anatomically complex diseases, such as pediatric craniosynostosis. Craniosynostosis represents a relatively rare (1 in 2,100–2,500 births) skull deformity due to premature ossification of one or multiple cranial sutures (16, 17). Since craniosynostosis may restrict the growth of the brain and can lead to deformation of the brain tissue due to overgrowth of the sutures and intracranial pressure, it is associated with a higher risk of impaired cognitive development (18). Therefore, early detection and treatment between 2 and 4 months for endoscopic and 6–9 months for open surgery are essential (17, 19).

In complex syndromic cases as well as in the planning of operations using image-based 3D modeling with computer-aided design (CAD) and computer-aided manufacturing (CAM) of implants used to create surgical templates, CT imaging and 3D reconstruction is necessary (20, 21). Therefore, 3D visualization is beneficial to account for craniosynostosis’ anatomical complexity and variability, but there is still little research into the pedagogical benefits of 3D VR models over traditional learning methods (8, 22, 23). The main aim of this study is to analyze the impact of virtual reality (VR) on the education of medical students and personnel by creating a VR database of a relatively rare pathology and examining how it influences the learning experience. Additionally, the study endeavors to contextualize the concept based on current literature.

## 2 Materials and methods

### 2.1 Data preparation

This educational development project included all craniosynostosis cases with available preoperative computed tomography (CT) imaging that were surgically treated at the Department of Pediatric Neurosurgery at the Children's Hospital in Basel from 2012 to 2022. Initially, data from 43 potential patients was screened. In 5 cases with imaging done in referring clinics, imaging could not be retrieved through data transfer, and in 1 case, data was damaged and unusable for further processing. Finally, we included 37 patients with multiple types of craniosynostosis and full CT imaging for further VR reconstruction.

### 2.2 Creation of the VR models and database

Preferably thin sliced CTs were considered for further processing. After collecting the respective Digital Imaging and Communications in Medicine (DICOM) files, datasets were anonymized, encoded, and stored on an external, password-encrypted hard drive.

The VR models were prepared in close collaboration with the Center for Medical Image Analysis & Navigation (CIAN), a research group from the Department of Biomedical Engineering at the University of Basel. In an explorative phase, different types of volumetric models were created within Specto (Specto Medical, https://spectomedical.com) and deployed on a Windows PC (Razer Blade 17 2022, Intel CPU i7-12800H, 16GB DDR5 RAM, NVidia GeForce RTX 3080 Ti GPU, Windows 11).The software uses ray-marching-based direct volume rendering to visualize the datasets without the need for segmentation (24). Transfer functions are used to map the Hounsfield unit (HU) value of each voxel to color and opacity.

For this study, the focus was on rendering the skulls as realistically as possible with little interference from soft tissue. Therefore, a specific transfer function visualizing bones, typically between 300 and 3,000 HU (25), was created and applied to the training datasets.

The DICOM-Data was imported to Specto, and the previously saved transfer function was used as a starting point for all datasets. Afterward, small modifications were applied to account for individual differences. The cleaning process involved removing irrelevant structures with similar values on the Hounsfield unit scale, so the model depicts only skeletal elements of the skull. All the unrelated structures were merged into one mask layer and hidden, providing a more comprehensible and clear visualization.

An HP Reverb G2 headset offering a resolution of 2160p × 2160p per eye and a 90 Hz refresh rate was used for immersive visualization and its accompanying controllers for direct interaction with virtual objects, enabling a more intuitive and engaging user experience.

**Figure 1:**
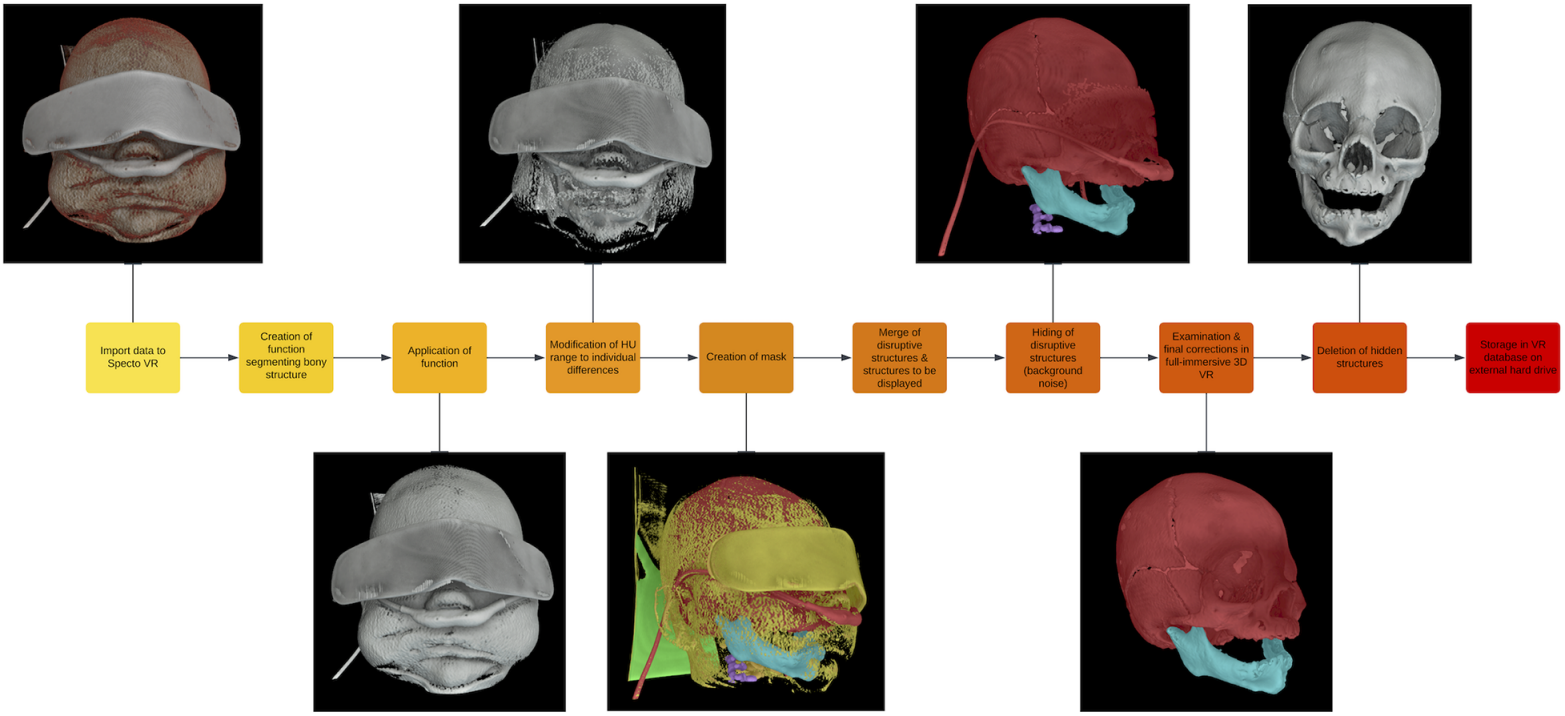
Workflow of the 3D VR model creation.

The models were then examined in VR, and the masks were retouched when necessary to ensure all relevant structures were visible. In the final step, the hidden structures were deleted from the volume to avoid false shadows on the model during the interactions in the 3D VR environment (Figure 1, Supplementary Video 1).

Lastly, the 3D VR models were stored systematically according to the type of craniosynostosis in the VR database on an external hard drive.

### 2.3 VR teaching and evaluation

**Figure 2:**
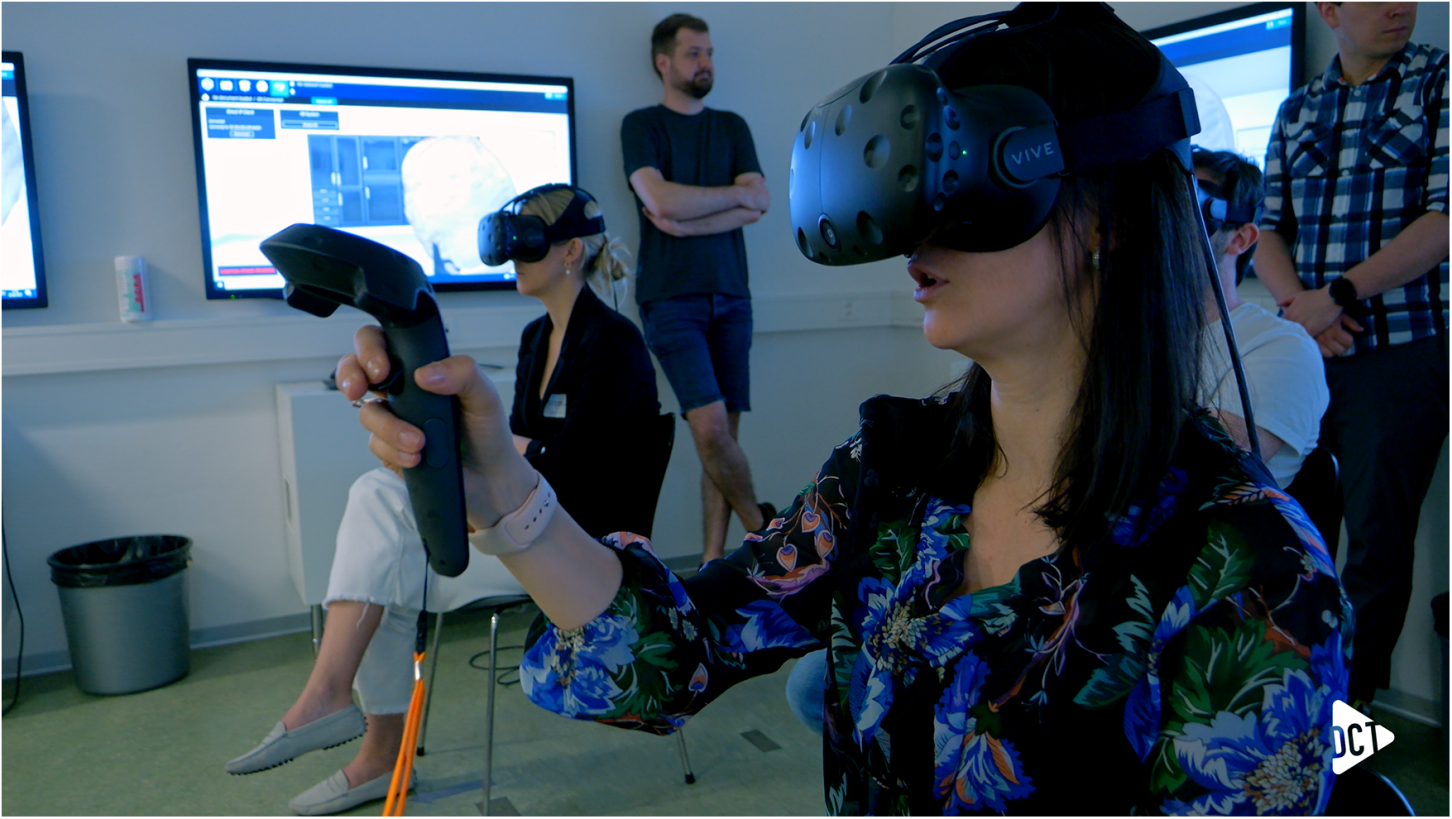
Participants with VR headset for immersive experience discussing the 3D craniosynostosis models.

Teaching was performed in a group setting, including a total of 17 participants, whereas 6 participants were simultaneously immersed in a multiplayer view, exploring and discussing the 3D model under the guidance of a host (Figure 2). Initially, the host guided the group through the model, highlighting anatomical particularities, whereupon the participants could move and manipulate the VR model individually without sharing the view. The sessions lasted 30 min, and three models were discussed. None of the participants had experience with VR teaching for medical purposes. The group consisted of resident surgeons (neurosurgeons and pediatric surgeons) and medical students representing a diverse range of medical experience within the group. The baseline characteristics of the participants in the teaching group evaluating the models included gender, age, medical specialty, and medical expertise, which are shown in Table 1.

**Table 1:** Baseline characteristics of the participants in the teaching group.

Number of participants	17
Gender	
Male	10 (58.82%)
Female	7 (41.18%)
Medical specialty	
Neurosurgery	12 (70.59%)
Pediatric surgery	1 (5.88%)
Medical student	4 (23.53%)
Mean age (years)	28.41 (± 6.83)
Mean medical experience (years)	3.59 (± 4.97)

**Figure 3:**
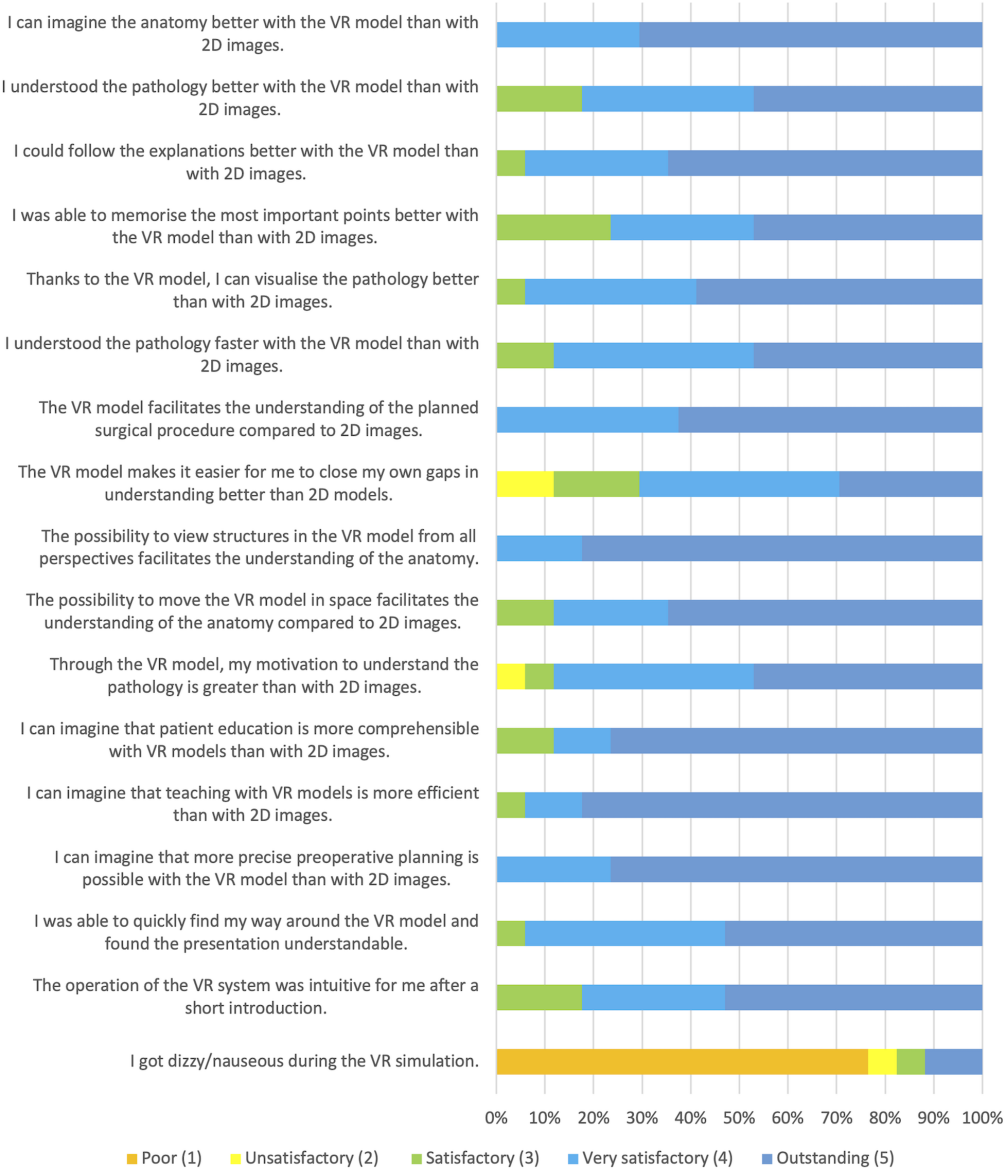
Results of the questionnaire.

After the teaching session, the experience was evaluated through a specially designed questionnaire handed to the participants (Figure 3). The assessment was performed on a Likert scale with five levels: poor (1), unsatisfactory (2), satisfactory (3), very satisfactory (4) and outstanding (5). The questionnaire considered different aspects critical to the learning process with the aim of determining whether teaching with VR improved them compared to learning using 2D images.

Primarily, we were interested in whether the 3D representation facilitates the understanding of the pathology and the foreseen surgical procedure and whether the clinical picture can be better imagined (Questions 5, 6, 11, 13 & 14). Furthermore, we assessed whether it was easier for participants to recognize and close their own gaps in understanding the anatomical nuances of the pathology (Question 12). Regarding the teaching process, we evaluated whether explanations could be followed better, whether visualization was more comprehensible, and whether the most critical points could be better remembered (Questions 7–9). Efficiency was evaluated by assessing subjective learning speed with 3D simulation (Question 10). As there are other areas besides teaching where the use of 3D simulations can be helpful, we wanted to know if participants could imagine that teaching would be more efficient, patient education more understandable, and preoperative planning more precise (Questions 16–18). Finally, the participants’ experience with the VR 3D simulation was explored, and any adverse side effects were assessed (Questions 19–21). As it is a new learning method, we wanted to know how it affects the participants’ motivation to understand the pathology (Question 15).

### 2.4 Systematic literature review

#### 2.4.1 Selection process

The systematic literature review was conducted by searching the databases PubMed and Embase up until the first of February 2024. Inclusion and exclusion criteria were defined using the population, intervention, comparison, and outcome protocol (PICO) (26). The primary search focus was “Teaching in VR in neurosurgery” while excluding all non-teaching topics such as surgical interventions or planning, review articles, non-full text articles or written in a language other than English. Since teaching craniosynostosis with VR is currently a very limited field, various neurosurgical topics were included, provided that the focus of the study was on evaluating VR education. A search string was built around the concepts “Neurosurgery”, “Education” and “Virtual Reality”. In total, 608 records (PubMed *n* = 190, Embase *n* = 418) matching our criteria were identified and included in the screening process. Fourteen were sought for retrieval, with two conference abstracts being excluded. During the eligibility assessment, 3 reports were excluded due to outdated techniques and/or missing education examination, and 9 were included in our systematic literature review. Details of the selection process are presented in the PRISMA flowchart (Supplementary Figure 1) (27).

#### 2.4.2 Summary of the studies’ content

The included studies analyzed the effect of VR on teaching in different areas of neurosurgery. The recent publication of the literature on this subject (2019–2023) reflects how topical and novel it is (Supplementary Table 1).

Five (55%) of the studies compared teaching effectiveness with VR models with traditional non-VR 2D learning materials. In 2019, Morone et al. compared learning with a 3D temporal bone model to 2D resources composed of illustrations from anatomy atlases and an online database, showing a subjective preference for the 3D model. The participants agreed on its higher educational value and potential to improve operative efficiency and safety (5). In 2021, Sugiyama et al. conducted preoperative planning sessions for patients with cerebrovascular disease using VR and evaluated subjective effectiveness compared to conventional imaging modalities with a questionnaire. To objectively assess the improvement in anatomical understanding, preoperative schematic illustrations were evaluated before and after the VR session, and it was found that the understanding of anatomy and the decision-making process improved (28).

Shao et al. in 2020, our group in 2021, and Ros et al. in 2020 conducted objective assessments of the teaching effectiveness of VR by dividing their study population into two groups, a traditional and a VR learning group. Shao et al. focused on skull base anatomy and showed that the results of the VR group exceeded those of the traditional learning modalities (4). Our group compared the time to aneurysm detection in neurosurgical residents and medical students using 3D VR compared to standard image visualization on a radiology monitor. No significant differences in detection time were observed within the resident group, but medical students were faster when using 3D VR (3). Ros et al. took a new educational approach by producing a 3D movie as an immersive tutorial on external ventricular drainage. They then assessed the students’ knowledge using a questionnaire and compared it with the results of the second group who studied a written technical note on the main surgical points. Knowledge retention was found to be significantly better with VR (29).

Instead of comparing VR's effects with 2D learning materials, Bairamian et al. used 3D-printed models. By investigating VR's educational potential and associated learning curve using aneurysm detection, they showed that it is easier to find small aneurysms, particularly with VR, than with 3D-printed models and that VR models’ subjectively perceived learning potential predominates (7).

Carlstrom et al. in 2022, Gonzalez-Romo et al. in 2023, and Atli et al. in 2021 did not directly compare the learning effect of VR with other educational modalities but developed immersive learning programs. Carlstrom et al. developed an online VR learning tool for the selection of craniotomy focusing on skull base tumor cases. For each VR model, a questionnaire was created with a selection of approaches for the trainee to choose from (10). Gonzalez-Romo et al. created an interactive virtual platform that simulates a neurosurgical anatomical dissection laboratory with VR models from high-resolution cadaver dissection photos. It enables real-time collaboration, creating a virtual meeting space. The system has been validated by neurosurgeons, who all agreed that virtual cadaver courses benefit learning 3D anatomy (30). Atli et al. evaluated the impact of a one-year course for medical students in which neurosurgical procedures, pathologies and neuroanatomy were taught using an interactive and immersive VR system. The levels of subjective competence were found to have increased for all the qualities assessed, and all the students felt that VR illustrated anatomical and surgical understanding was better and more memorable (31).

## 3 Results

### 3.1 Study population

In our teaching evaluation, a total of 17 participants were included. The participants were registered at the 6th Pediatric Neurosurgery Symposium in Basel 2023. There were no specific inclusion criteria, as the VR teaching was a part of the Hands-On session, and all registered participants were included. The group consisted of 4 (23.53%) medical students and 13 resident surgeons, of which 12 (70.59%) were neurosurgeons and 1 (5.88%) was a pediatric surgeon. The range of professional experience extends from 0 to 20 years with a mean of 3.59 ± 4.97 years. The mean age was 28.41 ± 6.83 years. Regarding the gender distribution, slightly more men were represented with 58.82% (*n* = 10) (Table 1).

### 3.2 Results of the questionnaire

The questionnaire results showed very satisfactory scores (mean 4.49 ± 0.25) across all evaluated qualities. The presentation of the VR models was easily comprehensible, the participants could quickly navigate the model (mean 4.47 ± 0.62), and the operation of the VR system was intuitive after a short time (mean 4.35 ± 0.79). The 17 participants attested to an enhanced understanding of pathology (mean 4.29 ± 0.77) as well as surgical procedure (mean 4.63 ± 0.5) with the 3D VR models. In addition to increased comprehension, the ability to imagine anatomy was better (mean 4.71 ± 0.47), and it was easy for the participants to recognize and close their own understanding gaps (mean 3.88 ± 0.99). Regarding the teaching process, the visualization of the pathology was comprehensible (mean 4.53 ± 0.62), and the explanations could be easily followed using 3D VR models (mean 4.59 ± 0.62). Since the participants reported that memorizing the most important points of the teaching was easily achievable (mean 4.24 ± 0.83) and comprehension was fast (mean 4.35 ± 0.7), the efficiency of the learning process increased.

Most of the participants could imagine that teaching is more efficient (mean 4.76 ± 0.56) with 3D VR models and agreed that patient education could be more understandable (mean 4.65 ± 0.7) and preoperative planning more precise (mean 4.76 ± 0.44). Motivation to learn about the pathology, another important factor in the learning process, was also perceived as improved by most of the participants (mean 4.29 ± 0.85).

The participants agreed strongly that the possibility of contemplating structures in the VR model from all perspectives, from all directions, from outside the model as well as from inside (mean 4.82 ± 0.39) and moving the model independently in the virtual space (mean 4.53 ± 0.72) helped to understand the individual anatomy. In 23.5% (4) a temporary feeling of dizziness occurred to some extent, but none of them had to exit the simulation earlier (Figure 3).

To examine whether the participants’ ratings varied by experience, we performed an ANOVA based on their years of experience. Analysis did not reveal significant differences in most of the ratings, indicating consistent satisfaction regardless of the level of experience. However, the question on preoperative planning precision showed significant variation (*p* = 0.018), suggesting that more experienced participants rated this aspect higher.

## 4 Discussion

Our study analyzed how 3D VR models of craniosynostosis could improve the subjective learning experience for neurosurgical education of medical personnel compared to conventional anatomical teaching. In all the qualities evaluated, our defined threshold of 3 (satisfactory) out of 5 for successful results was exceeded, verifying that the entire teaching process, consisting of understanding, efficiency, and motivation, was subjectively improved while using VR as a teaching method. The possibility of contemplating structures from all perspectives helped to follow explanations more easily and form a deeper understanding of the complex anatomy. Furthermore, participants stated that VR could be a great asset in patient education and preoperative planning. 23.5% (4 participants) reported temporary VR-related dizziness, but not to the extent that they had to leave the simulation.

In neurosurgery, it remains important to introduce new techniques that improve the understanding of complex and rare anatomical conditions. We chose the pathology of craniosynostosis for our models because it is a highly variable and rare entity, which makes 3D visualization essential. Little standardized anatomical teaching material is available for these rare pathologies (4). However, it should also be mentioned that depending on the type of craniosynostosis, a preoperative CT scan is not always necessary in today's clinical practice. Since we could only include patients with available preoperative CT scans, the frequency of different types of craniosynostosis in our study does not represent the frequency in the population. Our study population consists of 5.41% (*n* = 2) sagittal, 51.35% (*n* = 19) metopic, 40.54% (*n* = 15) coronary synostosis, and 2.70% (*n* = 1) pansynostosis, of which five were syndromic cases, representing anatomically complex variants. Compared to other cases, sagittal synostosis is the most common form in the general population, with around 60%. This is followed by coronal synostosis with around 25%, metopic synostosis with 15%, and lambdoid synostosis with 2% (32, 33), indicating that planning surgery for sagittal craniosynostosis in particular can be performed without CT imaging and thus without radiation exposure.

Traditional teaching methods in medicine usually consist of instructional modalities such as textbooks, illustrations, lectures, anatomical models, and cadaver dissection. Often, they cannot provide a truly immersive educational experience with a simple illustration of anatomical relationships, particularly in the complex field of neuroanatomy (4). In routine clinical practice, a patient's individual anatomy is usually visualized using 2D imaging such as CT or magnetic resonance imaging (MRI). Although the images are high quality with current technology, it can be difficult to understand the complex 3D craniocerebral anatomy with 2D images (34). The dissection of cadavers represents the gold standard in three-dimensional anatomical relationships and, therefore, serves to deepen the students’ understanding and train their practical skills, but its practical implementation is limited (31, 35, 36). In craniosynostosis, most patients reach adulthood, which is why there are no cadavers on which the pathology could be studied. Even if there were, autopsy could not be repeated or standardized and it would be difficult to transfer this approach to clinical cases. In addition, there would be qualitative shortcomings because the tissue of cadavers differs considerably from that of living tissue and can be distorted by the preservation process. Work on cadavers is known to be very time-consuming and associated with high costs (31, 35, 36). Some of these problems can be avoided by using 3D-printed models. Today, it is possible to produce individual and realistic models with high fidelity, but also with the limitation of considerable costs (37).

The main advantage of VR models is the accurate and immersive experience they provide, for any patient-specific anatomy. The user can manipulate the model in a virtual space, including zooming in and out, contemplating it from every angle, and even diving into the model and studying the anatomy inside to understand the three-dimensional relationships. Further information beyond the objective anatomy, such as functional imaging (i.e., fiber tracts, fMRI), can be visualized. As it depicts individual anatomy like CT and MRI scans but is more intuitive to grasp, it can simplify and speed up understanding in routine clinical practice and is, therefore, more efficient (29). Three-dimensional anatomical relationships can be studied by cadaver dissections. Still, VR offers the additional benefit of depicting the anatomy in layers (i.e., removing soft tissue selectively) and can be standardized and used repeatedly. The technical challenges associated with VR encompass hardware limitations, software integration, and compatibility, all of which directly impact the realism of the simulation. To ensure a smooth VR experience, it is crucial to employ high-end VR systems with high-resolution displays, precise tracking, and ergonomic controllers to minimize user discomfort and deliver immersive experiences. Routine maintenance and timely updates of these systems are necessary to ensure their reliability and keep them up to date. Investing in quality hardware as an initial investment and ensuring continuous development and seamless software integration is necessary to overcome these technical challenges. The latter represents the running costs after the initial outlay for the system. Reducing technical friction allows for an immersive experience closely intertwined with the learning curve. As users become more proficient with the technology, reducing frustration and increasing confidence, they are more likely to overcome the learning curve, making the transition to VR-based training smoother and more effective.

When comparing the costs and benefits of VR training, the initial costs for hardware, software, and development are complemented by the maintenance and upgrade costs over time. The initial integration of the technology and teacher training must also be considered. For example, a single human cadaveric head can vary from approximately $600 to $1,400 (38), while Mladina et al. describe a cost of $1,520 for a single resident to train (39). In addition, substantial expenses for maintaining an experimental laboratory *in vivo* must be considered (40, 41). Further benefits such as the possibility of repetitive practice and teaching, scalability, and the option for remote training underscore the efficacy in terms of cost-benefit.

The playful character of discussing cases in simulation and interacting with models improves the student's motivation, increases productivity and knowledge retention, and thus improves the quality of teaching for both students and teachers (8). Clinical cases can be quickly rendered and presented with comprehensive preoperative, intraoperative, and postoperative information, encouraging group discussions and critical thinking. This active engagement through exploration and feedback fosters independent learning (42). This active engagement through exploration and feedback fosters independent learning. Additionally, VR models promote teamwork and reasoning, with applications that extend to joint surgical strategy discussions and more transparent patient explanations (8).

Furthermore, since the models are digitalized, simultaneous collaborations could take place regardless of location, an advantage over physical teaching (i.e., 3D print models). VR models can be shared with other locations or countries, for example, via a cloud-based solution so that access to training is guaranteed and can be standardized through collaboration. Gonzalez-Romo et al. have proven this is technically possible with their cloud-based virtual platform. The simulated neurosurgical anatomical dissection laboratory allows for the individualization of the virtual space by storing the VR models in cloud storage. It enables real-time collaboration while working with them. Although the study was conducted remotely, participants received immediate feedback from experts during the learning sessions, giving this digital experience the essential social component of location-independent education (30). Shattuck et al. developed a similar platform for visualizing neuroimaging data in VR. Here, too, several users can work simultaneously in the same virtual space and discuss and interact with the models, opening up new possibilities for training and collaboration (43). Our program SpectoVR has been validated in such a scenario by Maloca et al. with three people in different locations across Europe meeting in the same VR space (44). The decentralization of education will facilitate international cooperation and simplify access to education in the future. VR platforms can play a key role, so further research is needed.

### 4.1 Study limitations

This study has several limitations that need to be mentioned. The questionnaire results are subjective data, and no objective assessment of knowledge retention was performed, nor was a direct comparison with other learning techniques such as 3D models or 2D images. Additionally, due to the novelty of the topic, we used a custom, non-validated questionnaire, as there are no standardized or validated questionnaires for this topic in the literature. Due to the small number, diverse backgrounds, and varying knowledge of participants, the results may not be representative of the entire population. Furthermore, since participants were enrolled with their own motivation and interest, there is a selection bias that could also have improved subjective results. Not only the number of participants was limited, but also the number of patients for certain types of synostosis, in particular sagittal synostosis. Future studies should include larger samples and divide them into randomized groups to investigate a direct comparison of objective learning outcomes between 3D VR and 2D instructional modalities.

## 5 Conclusion

Establishing a 3D VR database for VR-based anatomy teaching in craniosynostosis can improve and speed up the understanding and memorization process, increase motivation for the study process, and allow more efficient learning. It is cost-efficient and does not require significant additional resources or time.

## Data Availability

The original contributions presented in the study are included in the article/Supplementary Material, further inquiries can be directed to the corresponding author.

## Supplementary material

Supplementary Figure 1 PRISMA flowchart with details of the selection process for the systematic literature review.

Supplementary Table 1 Overview of the papers included in the systematic literature review.

Supplementary Video 1 Example video for a craniosynostosis model in SpectoVR.
