# Supplementary figures and images for: Enhancing educational experience through establishing a VR database in craniosynostosis: report from a single institute and systematic literature review
Source: Front Surg. 2024 Sep 4;11:1440042. doi: 10.3389/fsurg.2024.1440042 (PMC11408475; doi:10.3389/fsurg.2024.1440042)

### Identification of studies via databases and registers

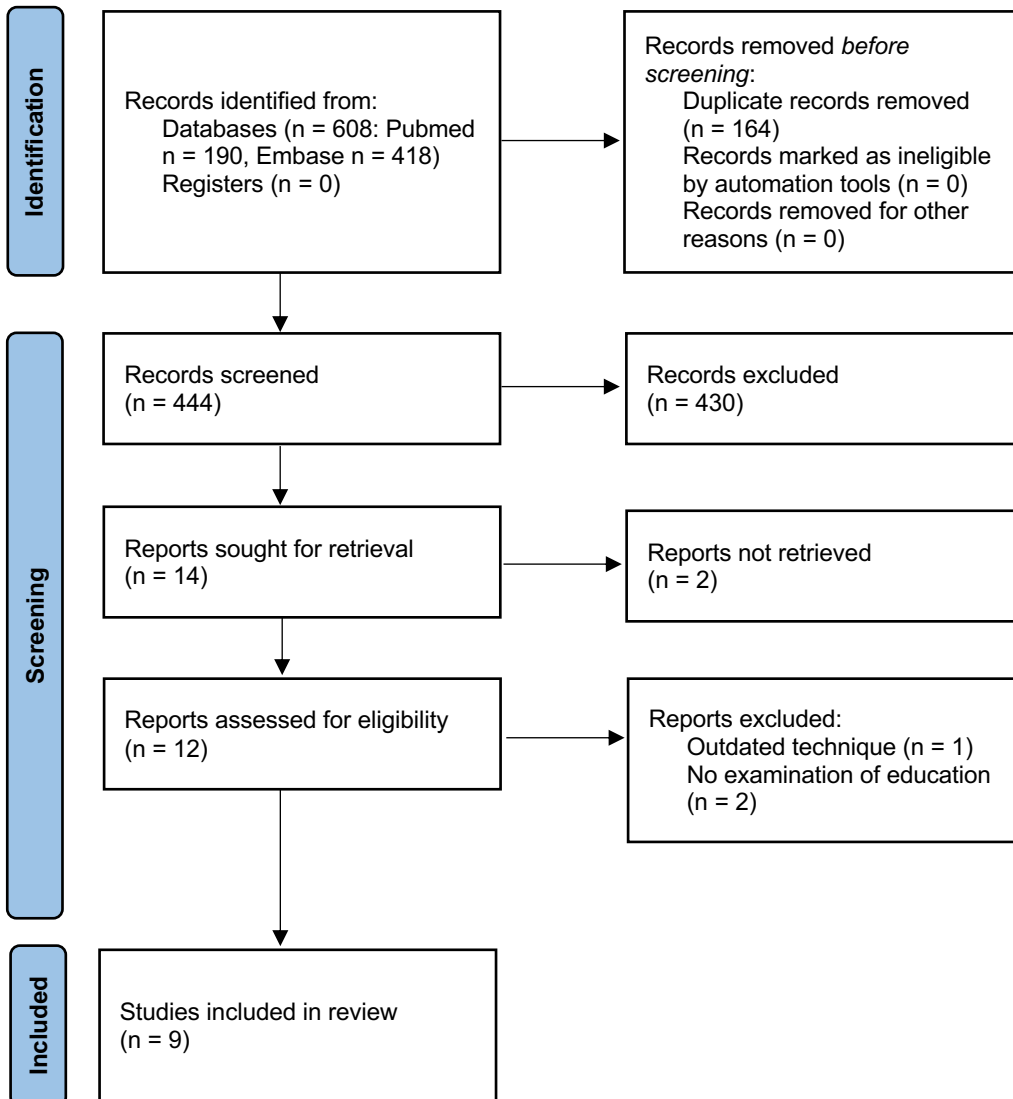

Supplement: Supplementary Figure 1 [file Image1.pdf]
