# Supplementary material for: Enhancing educational experience through establishing a VR database in craniosynostosis: report from a single institute and systematic literature review
Source: Front Surg. 2024 Sep 4;11:1440042. doi: 10.3389/fsurg.2024.1440042 (PMC11408475; doi:10.3389/fsurg.2024.1440042)
Supplement: Supplementary Table 1 [file Table1.docx]

| **Paper** | **Date of Publication** | **Journal** | **Authors** | **Institution, Country** |
| --- | --- | --- | --- | --- |
| Virtual, 3-Dimensional Temporal Bone Model and Its Educational Value for Neurosurgical Trainees | 2019 | World Neurosurgery | Morone et al. | Vanderbilt University Medical Center, USA |
| Immersive 3-Dimensional Virtual Reality Modeling for Case-Specific Presurgical Discussions in Cerebrovascular Neurosurgery | 2021 | Operative Neurosurgery | Sugiyama et al. | Hokkaido  University Graduate School of Medicine, Japan |
| Virtual reality technology for teaching neurosurgery of skull base tumor | 2020 | BMC Medical Education | Shao et al. | Yi-Ji Shan Hospital, Wannan Medical College, China |
| Randomized study comparing 3D virtual reality and conventional 2D on-screen teaching of cerebrovascular anatomy | 2021 | Neurosurgical Focus | Greuter et al. | University Hospital of Basel, Switzerland |
| Applying an immersive tutorial in virtual reality to learning a new technique | 2020 | Elsevier Masson | Ros et al. | Montpellier University, France |
| Virtual Reality Angiogram vs 3-Dimensional Printed Angiogram as an Educational tool-A Comparative Study | 2019 | Neurosurgery | Bairamian et al. | Nepean  Hospital, The University of Sydney, Australia |
| Three-Dimensional Modeling for Augmented and Virtual Reality-Based Posterior Fossa Approach Selection Training: Technical Overview of Novel Open-Source Materials | 2022 | Operative Neurosurgery | Carlstrom et al. | Mayo Clinic, USA |
| Virtual neurosurgery anatomy laboratory: A collaborative and remote education experience in the metaverse | 2023 | Surgical Neurology International | Gonzalez-Romo et al. | Barrow Neurological Institute, USA |
| A Comprehensive Multicomponent Neurosurgical Course with use of Virtual Reality: Modernizing the Medical Classroom | 2021 | Journal of Surgical Education | Atli et al. | University Hospitals Cleveland Medical Center, Case Western Reserve University School of Medicine, USA |
